# Local Bearing Estimation for a Swarm of Low-Cost Miniature Robots

**DOI:** 10.3390/s20113308

**Published:** 2020-06-10

**Authors:** Zheyu Liu, Craig West, Barry Lennox, Farshad Arvin

**Affiliations:** 1 Swarm & Computational Intelligence Laboratory (SwaCIL), Department of Electrical & Electronic Engineering, The University of Manchester, Manchester M13 9PL, UK; zheyu.liu@student.manchester.ac.uk; 2 Bristol Robotic Lab, University of West England, Bristol, Bristol BS16 1QY, UK; Craig.West@uwe.ac.uk; 3 Robotics for Extreme Environment Laboratory (REEL), Department of Electrical & Electronic Engineering, The University of Manchester, Manchester M13 9PL, UK; barry.lennox@manchester.ac.uk

**Keywords:** swarm robotics, range and bearing, sensory system, miniature mobile robotics

## Abstract

Swarm robotics focuses on decentralised control of large numbers of simple robots with limited capabilities. Decentralised control in a swarm system requires a reliable communication link between the individuals that is able to provide linear and angular distances between the individuals—*Range & Bearing*. This study presents the development of an open-source, low-cost communication module which can be attached to miniature sized robots; e.g., *Mona*. In this study, we only focused on bearing estimation to mathematically model the bearings of neighbouring robots through systematic experiments using real robots. In addition, the model parameters were optimised using a genetic algorithm to provide a reliable and precise model that can be applied for all robots in a swarm. For further investigation and improvement of the system, an additional layer of optimisation on the hardware layout was implemented. The results from the optimisation suggested a new arrangement of the sensors with slight angular displacements on the developed board. The precision of bearing was significantly improved by optimising in both software level and re-arrangement of the sensors’ positions on the hardware layout.

## 1. Introduction

Autonomous mobile robots are capable of handling complex tasks in unknown environments. Their precision and performance make them suitable in many applications, including industrial automation, security systems, extreme environments and agriculture. Nevertheless, with the continuous expansion of robotic applications, a single mobile robot cannot effectively fulfil tasks in large sized environments, e.g., autonomous farming [1]; hence, multi-robot systems that benefit from the collaboration and coordination of groups of robots offer significant potential in such environments [2].

Swarm robotics [3] is defined as a team of simple robots attempting to complete a global task by exploiting their local interactions. Among the multi-robot approaches, swarm robotics gives particular emphasis to features such as flexibility, scalability and robustness [4], which provide considerable advantages when compared to single robot and other multi-robot approaches. In general, swarm systems take inspiration from the social behaviours of animals, e.g., distributed and decentralised controllers, which are beyond the capabilities of individuals [5,6]. Each robot in a swarm is an autonomous individual with all the functionality of a mobile robot, and it collaborates with its neighbouring robots to solve a given task. Therefore, precise and reliable local communication and position estimation of neighbouring robots, in terms of distance and orientation, are essential to maintaining the group behaviour [7].

The purpose of the work described in this paper was to develop a simple, low-cost module for use in a swarm robotic system that would utilise a novel sensory and modelling approach that would provide localisation information to all individual robots within the swarm. The positioning system comprises a sensor and corresponding mathematical model that determines the robot’s bearing, defined as the relative angular position of neighbouring robots. The module was developed for the *Mona* robot [8] which is an open-source miniature robot for swarm robotics applications. This paper presents three important contributions: to begin, a mathematical model of *bearing* is proposed for this module. The model estimates the neighbouring robots’ angular positions by taking into account the surrounding sensory readings. Secondly, to improve the accuracy of the model, the parameters used within the mathematical model were modified using a genetic algorithm (GA) and its subsequent performance was tested, and finally a new layout of the communication module and the placement of its sensors were investigated. This was found to further reduce the positioning error produced by the system.

The remainder of this paper is organised as follows. Section 2 contains a review of work in the literature related to mainstream positioning techniques for common robots (Section 2.1), sensor systems for miniature robotic positioning (Section 2.2) and optimisation methods for positioning (Section 2.3). Section 3 introduces the proposed bearing model. Section 4 describes the optimisation algorithm and elaborates on the specific optimisation process. Section 5 introduces the experimental setup and describes it in detail. Section 6 analyses the results that were obtained from extensive testing and illustrates how the generic bearing model can improve the precision of the positioning system. Section 7 provides conclusions and suggestions for future research.

## 2. Related Works

A review of the most common positioning systems is presented in this section with a specific focus on solutions for miniature robots used for swarm robotic applications.

### 2.1. Range and Bearing in Robotics

Positioning approaches mainly consist of two components: one is the signal classification and the other is a localisation algorithm. The localisation algorithm allows robots to employ an appropriate method to estimate their current positions relative to other robots in a group. It includes geometric positioning, proximity perception and vision analysis algorithms. In real-world applications, the prerequisite of autonomous motion requires the robots to acquire precise location information [9]. Common positioning methods rely on various internal sensors, such as gyroscopes [10], photoelectric encoders [11] and multi-spectral instruments which can be fused to provide accurate estimates of the robots’ positions. Nevertheless, these conventional methods can be inaccurate because of defects related to these internal sensors [12], and unknown uncertainties in, for example, the offset of the wheels and vibration of the robot’s mechanical structure. Such errors will accumulate and be amplified, potentially introducing significant errors in the position estimates of the robots. To resolve such problems, an external positioning reference without any accumulative error can serve as the independent third party to provide location information, which can then be exploited to correct the robots’ positions [13]. The most common external localisation reference comes from the Global Positioning System (GPS) that requires a direct line-of-sight communication to the satellites. Hence its transmission signal may have poor coverage and intensity, particularly in indoor or subterranean environments [14]. Therefore, indoor positioning technologies using alternative technologies that provide an external reference have been proposed to tackle the problem of accurately localising mobile robots. This include the use of technologies such as wireless local area network (WLAN), radio-frequency identification (RFID), ZigBee, Bluetooth, ultra-wideband (UWB), infrared (IR), computer vision (CV) and light detecting and ranging (LiDAR).

Due to the short response time and high accuracy, IR positioning systems are widely used to localise robots. In [15], an innovative IR sensory system was proposed to position a mobile robot in an area, so-called an *intelligent space*. By measuring the differential phase-shifts of a sinusoidally modulated infrared signal transmitted from a robot, the differential distance from the phase-shift data could be obtained. This information can then be analysed using hyperbolic trilateration to obtain an estimate of the position of the robot. With appropriate specification of the system parameters, the accuracy of the positioning of this technique has been shown to be in an acceptable range with an error of less than 10 cm. In [16], the combination of IR sensors and a monocular camera was shown to provide a robust estimate of robot pose, with the technique able to fuse information from different sources. The primary innovation with this technique was that it was able to exploit the information from the camera to compensate for the relatively poor estimate of the robot’s position that was obtained from the IR scanners, and also resolve problems related to the relatively slow vision analysis systems through frequent updates from the IR sensors.

RFID is a mature and reliable technology that applies wireless communication to automatically identify objects. The implementation of RFID technology requires the use of an RFID reader equipped with one or more antennas and active or passive transceivers [17]. In [18], mobile robots carried an RFID reader at the bottom of the chassis, which was able to read RFID tags on the ground to enable it to provide an estimate of its position. The paper proposed a novel triangular pattern for arranging the RFID tags on the floor to reduce the estimation error that is obtained using a more conventional square pattern.

Ultra-wideband (UWB) is a wireless communication technology that emerged from time-domain electromagnetic technology. The UWB has been utilised for positioning of mobile robots in various applications. In [19], an obstacle avoidance method for an autonomous unmanned boat was presented. The UWB positioning system was applied to monitor and transmit the real-time position of the unmanned boat simultaneously. The UWB distance estimation was also utilised in multi-robots formation control [20]. In general, the positioning system based on the UWB is similar to radio frequency localisation system that consists of fixed anchors and mobile antennas. In addition, the associated software methods can be developed with various techniques, such as distance estimation with wave reflection, active measurements based on time of flight (ToF) [21], time of arrival (ToA, TDoA) [22], angle of arrival (AoA) and fingerprinting (RSSI mapping), and their combinations [23]. An improved positioning performance for *Pozyx*, a low-cost commercial positioning system, was proposed in [24]. The method used a modified multilateration algorithm with adding a number of anchors as well as adjusting their positions. The results demonstrated an improvement in localisation precision by approximately 40%.

Ultrasonic waves can be used to determinate distance between a fixed station and the mobile targets. In ultrasonic positioning systems, the pivotal operation requires multiple ultrasonic sensors on-board to receive ultrasonic waves [25]. A representative example associated with positioning of mobile robots using ultrasonic-based system is provided in [26]. In this work an indoor ultrasonic positioning system with four beacon nodes was proposed. It was able to determine the position of an unknown node in real-time. The positioning principle exploits the time difference between the ultrasonic sensor and RF transmission. Hence, it calculates the distance from the time gap find the unknown node’s coordinates. There are several research works that combined ultrasonic systems with RF technology to estimate the positions of mobile robots [27,28]. In [27], the indoor positioning system referred to as Cricket comprised receivers that were mounted on the robots and location beacons that were attached to the ceiling which continuously transmitted ultrasonic and RF signals. The limitation of this method is that it requires considerable manual configuration of the ultrasonic sensors to achieve coverage of large spaces, which would increase the positioning cost. The improved hybrid positioning method proposed in [28] addressed this problem by significantly reduce the required configuration. The positioning system was similar to cricket, with the objects’ position being estimated using trilateral algorithms. The novelty with this approach was that it employs hop-by-hop localisation which requires the precise location of some nodes in advance; however, the others can be automatically located in real-time.

Visual localisation has recently become the mainstream positioning method for relatively large mobile robots operating in complex environments [29]. In such systems binocular depth cameras act as sensing devices, providing information about the environment which can then be used to provide an indication of a robot’s position within it. In [30], a multi-fisheye camera system was proposed that was able to provide 3D perception for self-driving cars. The localisation method involved in this work required the correspondence between 3D space map and 2D images obtained from a camera system to be determined. The algorithm used to determine the correspondence [31] was based on Plücker coordinates which solves the pose estimation problem in two steps: (i) solve for the depth and (ii) solve for the rigid transformation with absolute orientation. Results obtained using GPS ground truth testing verified that the camera-based system could acquire good performance in the localisation and pose estimation. In related work, a modified mobile robot localisation approach was developed that was based on classification with a rejection option using computer vision [32]. This approach employed topological map information, based on supervised learning, to optimise the performance of the localisation and navigation tasks in mobile robots. The methodology comprised two core components after capturing the images: (i) feature extraction and (ii) classification. With respect to feature extraction, it considered standard methods in digital image processing. For classification, it applied machine learning methods with a rejection option. Compared with the classic localisation systems using an omnidirectional camera, the proposed method can provide higher accuracy rate (99.94%) and smaller computational time in consolidated feature extractions and machine learning techniques. It also performed well in navigation test, which verified that it increased the navigation efficiency and reliability in mobile robotics.

LiDAR positioning technology exploits the LiDAR sensor, whose basic principle is similar to radar, yet it adopts the invisible light rather than radar waves to detect the distance. The LiDAR system serves as a typical localisation system by emitting a laser beam and receiving the reflected signal to calculate the distance to an object. The position and velocity of a target can be found by fusing the information obtained from multi-sensors, which is commonly applied in autonomous navigation of vehicles [33]. In [34,35], looking at different situations of mobile robots, different LiDAR localisation methods were proposed. The work [34] developed a new algorithm to expand the crossover detection function by incorporating the crossover measurement from the robot’s perception and its relevant topological information with the pre-defined path network, and importing this information into the localisation system. The new method requires sufficient LiDAR data, and is mainly reliant on the search for the available free spaces that combine the obstacles occupied in setting grid with a Kalman filter used to data association and tracking. In [35], an effective and robust system was designed for mapping and localisation of the micro unmanned aerial vehicles (UAVs) in an indoor environment. The estimation of a UAV’s 3D position is derived through efficiently fusing measurement data from the primary and secondary LiDAR. The innovative method assembles the point cloud obtained from LiDAR with the inertial data measured by a simple inertial measurement unit (IMU) to integrate the 3D data set. Specifically, localisation is performed by exploiting a scan matching approach based on a customised version of the iterative closest point (ICP) algorithm, while mapping is achieved by extracting robust line features from LIDAR measurements.

### 2.2. Range and Bearing for Miniature Robots

In the choice of the best technology when designing a localisation system, considering the distinctive traits for each approach, it is indispensable to balance the trade-off among environmental conditions, user demands and performance parameters. The localisation techniques described above utilised sophisticated hardware and complex algorithms which would require substantial computational processing and storage resources [36]. All these aspects make it difficult to adapt the techniques to simple, micro-robotic platforms that can be scaled down in complexity and size [37]. Considering the features of miniature multi-robot systems—low-cost, small, low-power, simple in structure—the existing positioning systems need to be selected and slightly modified to adjust for miniature swarm robots.

For a micro-swarm robotic system, its self-positioning implementation needs to interact with robot members, This interaction process includes two core modules, perception and communication. The perception function mainly relies on the sensor’s performance and precision. Actually, the communication system can support the robotic system in continuously updating and exchanging the information from the sensors between each individual agent [38]. In the swarm robotic scenario, the implementation of perception also entirely relies on the inter-robot communication system, including the distance, bearing and velocity [39]. An inter-robot communication system based on multiple IR sensors is a suitable choice for a multi-miniature robots system, as IR sensors system are characterised by the high precision, low aperture angle, affordable sensors and the low power requirements, which properly match the miniature robot’s requirements [40]. In addition, IR radiation can not only be used to detect and perceive surroundings, but can also be specifically modulated to transport messages like binary phase shift keying and frequency shift keying. Except for peripheral unit, the robot’s main processor is also another pivotal component in calculating the bearing. The basic requirement for the main processor is to offer the constant change of individual behaviour in real-time, and have the capability to support the robot to participate in the swarm’s data exchange, to determine and share position information. Table 1 lists different miniature robotic platforms with similar multi-IR sensory systems but with different sensor configuration and hardware structure. There are inevitable errors in most of the positioning studies that are used in real-world applications.

### 2.3. Optimisation

There are various methods for calibrating errors in robotics, such as using artificial neural networks [53]; and bio-inspired optimisation such as ant colony optimisation (ACO) [54] and particle swarm optimisation (PSO) [55]. For example, a positioning approach using a modified ACO algorithm was developed in [56], enabling a multi-robot system to accurately approach an odour source. Apart from this, in [57], PSO was utilised to determine multi-robot positions in a football match. The results demonstrated the feasibility of applying the PSO algorithm to finding robots’ positions. In robotics for surgical operations, a model-free based deep convolutional neural network was proposed [58]. In another study [59], a PSO based backpropagation neural network algorithm to solve an inverse kinematics problem of a medical puncture surgery robot was proposed, which could achieve precise positioning with an error of less than 0.1 mm.

The genetic algorithm (GA) is a heuristic global optimisation algorithm which takes inspiration from the biological principle of natural selection. It was widely used for optimising various problems in robotic systems. In tuning PID control gains for a robotic arm [60], GA provided a better performance in parameter optimisation than conventional methods such as trails and error and empirical approaches. In another study [61], GA was used to identify unknown parameters for a model of an existing 7-DOF hydraulic manipulator. In a study on mobile robots localisation, GA was utilised to solve a self-localisation problem in an indoor environment [62]. GA has been used for various optimisation applications in robotic path planning [63], as a fuzzy controller for obstacle avoidance [64], for training a deep neural network [65] and for engineering manufacturing [66]. In a study on multi-robotics, a spatially structured genetic algorithm was proposed to improve the performance of positioning [67]. In the previous work on swarm aggregation [68], a GA was used for optimising weight parameters of the fuzzy system which estimates turning angles of the robots based on the captured sound amplitudes form four microphones. Therefore, the feasibility of using a GA optimisation in multivariable problems, e.g., in swarm robotics, has been demonstrated. GA also has the advantage that it does not get stuck at local optimum values, but searches the full population to find the global optimum. A disadvantage of using a GA is that it can be slower than alternative methods; however, as the optimisation was being done offline, speed was not a concern and the advantage outweighed this negative.

### 2.4. Summary

In summary, various localisation technologies, such as infrared, LIDAR, UWB, ultrasonics and RFID techniques, have been proposed and applied in real-world applications. For application in multi-robotic systems, the most suitable technology relies on a variety of factors, such as cost, size and required accuracy. For the low-cost, miniature swarm robotic system that is the focus of this work, a multiple infrared sensory system was believed to be the most suitable sensory system for the positioning. Compared to the previous studies, this work focuses on the provision of more precise bearing estimates for use with low-cost, simplistic robots.

There are many optimisation methods available to solve problems such as this, including particle swarm optimisation, differential evolution and genetic algorithms. In this work, we used a GA as an initial tool to determine the feasibility of the proposed approach. It is acknowledged that the most suitable optimisation method to use is problem dependent, and although GAs have been demonstrated to be robust and efficient, alternative approaches will be investigated in future research.

## 3. Bearing Model

To investigate the performance of the developed range and bearing hardware module and to improve the accuracy of the function, a bearing model to estimate the neighbouring robots’ relative angular distance was proposed.

### 3.1. General Bearing Model

**Figure 1:**
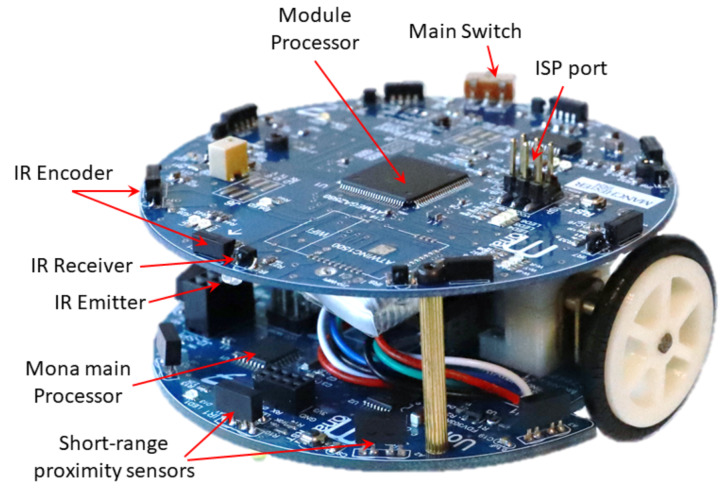
Mona robot equipped with the developed communication module.

For multi-robot systems, the common approach to determining the bearing to a neighbouring robot is to use the multi-sensory inter-robot communication system. Using this technique, each agent within the swarm possesses a communication module and those robots that receive a signal transmitted by one robot can infer the relative position (both range and bearing) of this robot through analysis of a physical property of the signal, such as signal intensity [14,40]. In this study, Mona robots [8] serve as the individual members of the robot swarm, and controlling the overall positions of these robots within the swarm is vital if cooperative tasks using many individual members are to be achieved. For communication purposes, as shown in Figure 1, each Mona is equipped with 8 high-precision IR sensors that act as a local communication module ensuring information is exchanged between the robots and hence allowing the relative position of each robot to be determined. In this study, estimation of the relative angular distance (bearing) of a neighbouring robot was investigated.

Mona perceives the IR intensity and translates it into a value that can be used to calculate the bearing based on the corresponding mathematical model. As Mona’s IR sensors are distributed symmetrically at fixed angles 45${}^{\circ }$, the relative angular position of the detected robot can be estimated using Equation (1) [8]:

(1) $$\phi =\arctan(\frac{\sum_{i=1}^{5}\hat{s_{i}}\sin\left(\psi_{i}\right)}{\sum_{i=1}^{5}\hat{s_{i}}\cos\left(\psi_{i}\right)}),$$

where ϕ is the estimated angular position; $\psi_{i}$ is the angular distance between ith sensor and the “front” orientation of the robot. $\hat{s_{i}},i\in \left\{1,2,3,4,5\right\}$ is the received IR intensity from sensor {IR${}_{1}\;Front$, IR${}_{2}$ *Front-right*, IR${}_{3}\;Right$, IR${}_{7}\;Left$, IR${}_{8}$ *Front-left*}, respectively. The arrangement of the IR sensors on the communication board is illustrated in Section 5.1.

Due to the sensor layout on Mona, only five IR sensors (receivers) participate in each bearing estimation with respect to the angular position of the neighbour. When the IR signal is transmitted from a neighbouring robot, only those sensors on the receiver Mona that are facing the emitting robot receive the infrared signal. The remaining three sensors located at the other side of robot do not receive the signal as it is blocked by the robot body. A Mona robot transmits an IR signal, and the neighbouring robot receives the IR signal with its five IR receivers. The receiver robot translates the different signal intensities obtained into digital values via the analog–digital converter (ADC), located on the micro-controller. With this information, the receiver robot is able to calculate the relative angular position of the other robot using the bearing model presented in Equation (1).

### 3.2. Proposed Bearing Model

The proposed method of positioning relies on Equation (1), which does not consider disturbances or errors. However, in any real-world system, there will be various sources of disturbance from the external environment that will introduce errors into the bearing estimate. For example, anything that emits heat will generate and transmit IR radiation. Therefore, the IR from the external environment (e.g., sunlight) will be an interference factor to the bearing system, which must be eliminated. Furthermore, the IR sensors have a narrow detection angle, which introduces errors in the measurement and hence discrepancies between the actual and estimated bearing.

To reduce the error and increase the accuracy of the bearing estimate, without increasing the cost of the hardware, Equation (1) is modified with the introduction of weights (gains) that are applied to the sensor measurements, $\hat{s_{i}}$. As discussed, two factors introduce errors into the bearing estimates: noise in the measurements of infrared intensity and the location at which the sensors are placed on the robot. The modified bearing model is shown in Equation (2) is presented below:

(2) $$\phi =\arctan(\frac{\sum_{i=1}^{5}\eta_{i}\hat{s_{i}}\sin\left(\psi_{i}\right)}{\sum_{i=1}^{5}\eta_{i}\hat{s_{i}}\cos\left(\psi_{i}\right)}),$$

where $\eta_{i}$ is the weight value for IR intensity of *i*th sensor. The values of the weights were determined by taking measurements of IR intensity relative to known robot bearings during a series of experiments.

To further reduce the error of bearing, weights were applied to the sensors’ angular distances on the board. Based in the original design, the angular distance between the IR sensors was 45${}^{\circ }$; however, we are proposing changes on the physical position of the sensors on the PCB to investigate further improvement on the bearing accuracy. Therefore, the resulting model is shown in Equation (3):

(3) $$\phi =\arctan(\frac{\sum_{i=1}^{5}\eta_{i}\hat{s_{i}}\sin\left(\lambda_{i}\psi_{i}\right)}{\sum_{i=1}^{5}\eta_{i}\hat{s_{i}}\cos\left(\lambda_{i}\psi_{i}\right)}),$$

where $\lambda_{i}\in \left[0,2\right]$ is the weight values for angular distance which will relocate the sensor position in hardware level and $\eta_{i}\in \left[0,2\right]$ is the weight values which are applied on the sensors’ reading in software level.

## 4. Optimisation of Bearing Model

**Figure 2:**
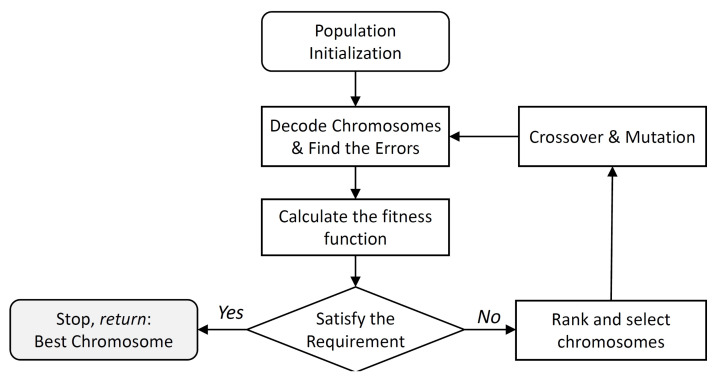
Flow chart showing the utilised optimisation process with the genetic algorithm.

As the analysis above describes, the bearing error is unavoidable in the estimation process and modification and potential optimisation of the bearing model is important if this is to be addressed. The optimisation of the bearing model presented in (3) can be considered to be a parameter optimisation problem without complex constraints. The only constraint is the value of the parameters which must lie between 0 and 2. GA is selected as the feasible optimisation method. The algorithm firstly sets randomly generated chromosomes containing the parameters which have to be optimised. It encodes each potential solution to a specific problem on a simple chromosome-like data structure. To guarantee continuous evolution, in each iteration, crossover and mutation operators are applied to a certain number of chromosomes form the population. In each generation, a chromosome’s individual suitability is evaluated such that the best individual is identified. This process is continued until a chromosome that satisfies the preset condition is selected [69]. The complete flow chart of the optimisation process which is used in this work is shown in Figure 2. In addition, the basic parameters of the genetic algorithm are illustrated in Table 2.

### 4.1. Initialisation

In this section the procedure for optimising the parameters is explained. The specific parameters that were optimised were the magnitude of the IR readings and the positioning of the IR modules on the board. These parameters are the five weights corresponding to the magnitude of the sensor readings from the five IR sensors, represented by $\left\{\eta_{1},\eta_{2},\eta_{3},\eta_{4},\eta_{5}\right\}$, and five weights associated with the angular distances between the infrared modules on the hardware board, represented by $\left\{\lambda_{1},\lambda_{2},\lambda_{3},\lambda_{4},\lambda_{5}\right\}$ in Equation (3). Therefore, the GA is required to optimise a total of 10 parameters.

**Figure 3:**
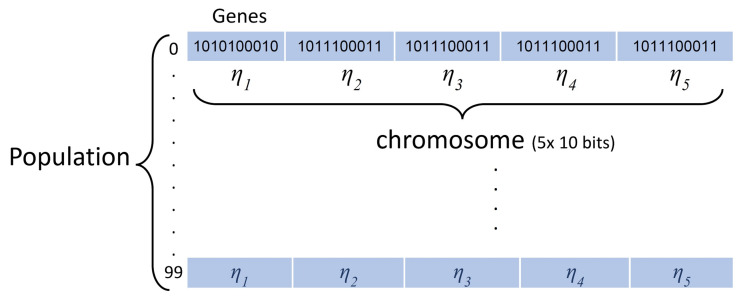
An example of population structure for Equation (2) with 5 parameters, $\eta_{1}\ldots \eta_{5}$.

The GA starts by initialising the population. Each population is identified as a collection of *chromosomes*, and each chromosome consists of discrete units called *genes*. The genes are expressed as binary numbers, as this simplifies the subsequent operations of crossover and mutation. The first generation of parental population is randomly initialised with subsequent generations specified based on the rules defined by the GA. The relationship between these three items is shown in Figure 3. A chromosome adopts the binary encoding method with 50-bit (5 × 10 genes). In the starting stage, the chromosomes are set to random binary values (0 s or 1 s). After the binary population is generated, a decoding function is used to find the decimal value of each parameter (weight).

### 4.2. Fitness Function Evaluation

The fitness function is an evaluation criterion used to compare the performances of individual solutions. The objective of the optimisation is to reduce the error between the actual bearing and the estimate provided by the model. Therefore, the fitness function is defined as below:

(4) $$J=\frac{\sum_{i=1}^{M}e_{i}^{2}}{M}\;,$$

(5) $$e_{i}=\varphi_{i}-\phi_{i}\;,$$

where $\varphi_{i}$ is the real bearing angle, $\phi_{i}$ is the estimated angle calculated by the model (Equation (1)) and *M* is the sample size of the experiment. In this experiment, the sample size is *M* = 400. Results were obtained from five IR receivers that received signals from the emitting robot and were placed at five different angles with distances of 5–20 cm (16 samples). Each experiment was repeated five times; therefore, we had *M* = 400 samples.

### 4.3. Selection

After each iteration, the parental chromosomes evolved as they might if they were subjected to a survival of the fittest process like in natural competition. Those chromosomes whose values of the fitness function are “better” have higher probabilities of passing on to the next iteration. A common selection operation, proportionate roulette wheel selection (PRWS) [70], is used to screen out the “excellent” individuals from one evolutionary cycle to the next. The probability that an individual in a population is selected is proportional to the value of the individual’s corresponding fitness function.

### 4.4. Crossover and Mutation

**Figure 4:**
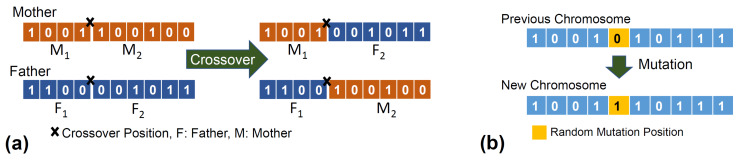
Examples of (**a**) crossover and (**b**) mutation operations for a single parameter (only 10 bits of a chromosome).

A series of operations is applied to the chromosomes to create new “child” chromosomes. In the crossover operation, a new chromosome is generated by exchanging part of the “father” chromosome with a corresponding part of the “mother” chromosome, which means that the offspring consists of the pre-crossover point section from the “father” chromosome followed by the post-crossover point section of the “mother”. The crossover position is selected randomly. The crossover probability is typically set to be relatively high to ensure the crossover operation does not occur sparsely. The crossover process for a single parameter (10 genes) is shown in Figure 4a.

In terms of mutation, a bit flip mutation is utilised to change the randomly selected genes in a selected chromosome from 0 to 1 or vice versa. A limited number of chromosomes are selected for mutation due to a lower probability, $p_{m}$. For the selected chromosomes, 10 bits (one bit for each parameter) are randomly selected for the mutation.

Mutation is introduced as it improves the searching capability of the GA. In addition, the mutation process is presented in Figure 4b. Crossover and mutation [71] were applied to ensure that search space was maximised and that the best solutions (chromosomes) survived.

## 5. Experiments

### 5.1. Hardware Platform

Mona (shown Figure 1) is an open-source swarm robotic platform which was developed as a low-cost mobile robot for research and education [8]. It was developed based on the Arduino (https://www.arduino.cc/) architecture, which makes the robot a versatile platform with full access to open-source libraries developed for Arduino projects. Mona has been utilised in many research projects with real-robot experiments; e.g., multi-robot system in control theory [72,73] and long-term autonomy of swarm [74]. The simulated models of Mona were also used in various projects; e.g., swarm flocking [75]. Although the robot is well established as a research platform for swarm robotics, its localisation issue limits the implementation of fully decentralised control scenarios. Therefore, a new communication module has been developed to enable local short-range communication and to increase local perception of the robot in a swarm scenario.

**Figure 5:**
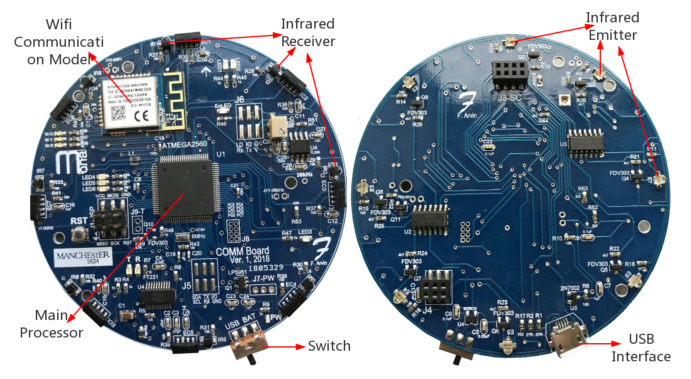
The top and bottom views of Mona’s communication module.

There are several functional modules/components arranged on the 8 cm diameter circuit board, the so called *communication board* (see Figure 5), which is attached on top of Mona robot. There are eight IR sensors installed on the perimeter of the communication board uniformly with angular separation of 45${}^{\circ }$. Each IR sensor system consists of three components, one emitting diode, one modulated receiver (encoder) and one photodiode. The Atmega 2560 micro-controller is placed at the centre of the board, which consists of several internal modules, including 16 ADC channels that are exploited to connect with the IR sensors to handle the signal and to monitor the condition of the battery, and 86 general purpose input/output ports (IOs). In addition, the micro-controller supports several serial communication methods, such as RS232, I^2^C and SPI that are utilised to program the flash memory or for communication with external modules. Both the top and bottom structure diagrams of the circuit board with IR sensors are shown in Figure 5.

In this project, we used C programming language to write the functions to manage IR emitters and receivers using AVR microcontrollers’ ADC channels and digital ports.

### 5.2. Experimental Setup

Based on the proposed bearing model, the angle estimation of neighbouring robots requires the magnitude of received IR at corresponding ranges. The angles calculated by the bearing model from the data are defined as estimated angles, ϕ. When running experiments, the initial angle to a neighbouring robot is defined as the real angle, φ.

**Figure 6:**
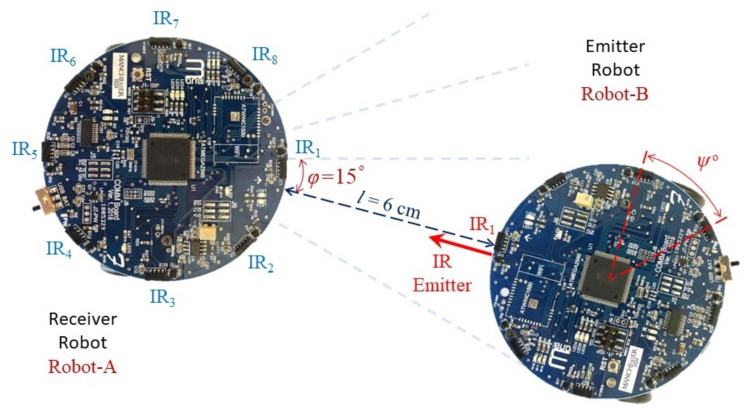
An example of the practical experiment where a transmitter robot is placed l=6 cm from the receiver robot with an angular distance of φ = 15${}^{\circ }$.

The experiment employed two Mona robots: Robot-A received the IR signal to calculate the neighbour’s angular position, and Robot-B, which was detected by Robot-A, transmitted the IR signal. Figure 6 shows an example of experiment where Robot-A is calculating the relative angular distance of the neighbour robot; i.e., Robot-B. In each measurement, five different readings of signal magnitude from the five IR receivers of Robot-A, ${\hat{s}}_{i}\;i\in \left\{1,2,3,4,5\right\}$, are recorded. This Table 3 shows the standard values of the variables used in the experiments.

## 6. Results

### 6.1. Experiment Results

**Figure 7:**
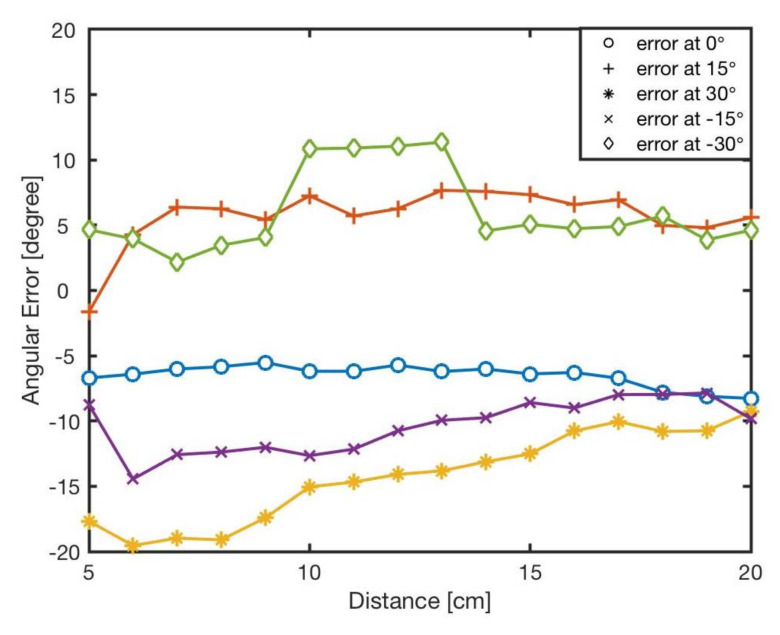
The bearing errors, *e*, at different angles in various distances, $l\in \left[5\text{–}20\right]$ cm.

**Figure 8:**
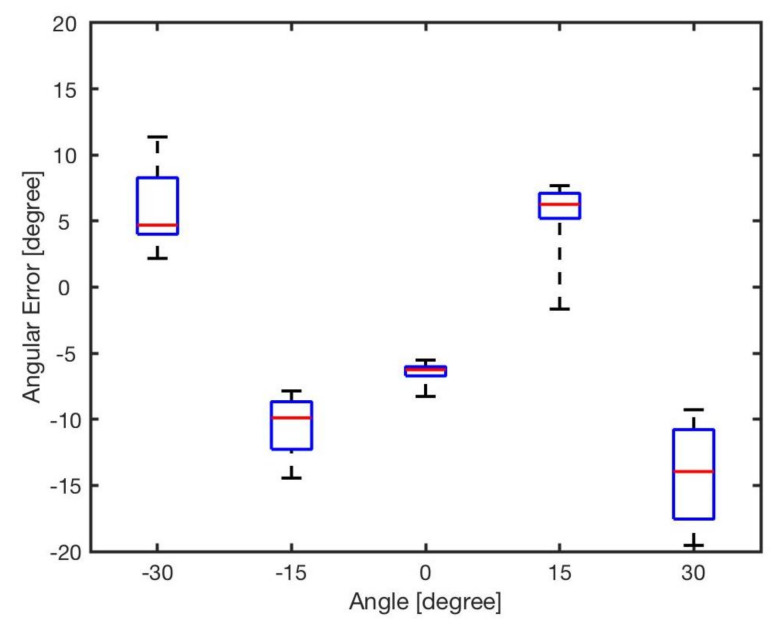
Angular error for different angles $\varphi \in \{-30^{\circ },-15^{\circ },0^{\circ },15^{\circ },30^{\circ }\}$.

The estimated angle, ϕ, was calculated by the bearing model Equation (1). Figure 7 shows the angular errors for different angles and distances. The results showed that increasing the distance between two robots resulted in reducing the error. This is because of the radiant intensity of the IR emitters and viewing angle of the receivers. Figure 8 reveals the angular error for different angles for in distances from *l* = 5 cm to *l* = 20 cm. As shown in the results there was no correlation between angular position of the neighbouring robot and the error in estimation. The error was mainly because of the robot’s heterogeneity of IR components in hardware level. Similar heterogeneity in robot sensory systems was also reported in [5,74].

Apparently, errors are inevitable, and all the errors almost exceed $5^{\circ }$, with the maximum value up to about $20^{\circ }$. For each angle, the maximal bearing errors are as high as $\left\{-8.27^{\circ },-7.68^{\circ },19.54^{\circ },14.45^{\circ },-11.36^{\circ }\right\}$ respectively. The average error is shown in Table 4. From the result, it can be concluded that the general bearing model cannot directly be used, and hence it needs to be optimised and modified.

### 6.2. Optimisation of Bearing Model

**Figure 9:**
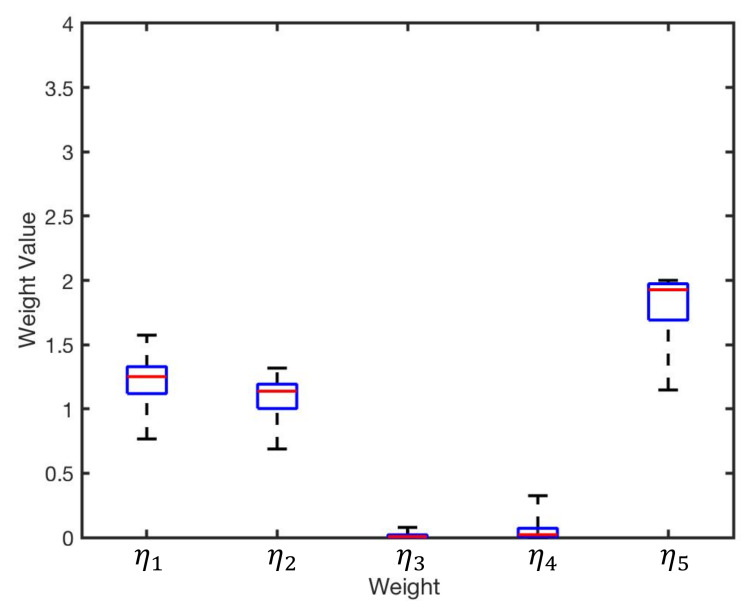
Distribution of the obtained results for $\eta_{1}\ldots \eta_{5}$ from 100 experiments.

GA was applied as an optimisation method to reduce the error related to infrared intensity. There is always the possibility of converging to a local optimum; hence, we repeated the experiments 100 times to avoid the issue. Figure 9 illustrates distribution of the obtained weights from the 100 experiments. The group of intensity weights with correspondence to best fitness value is identified as the optimum result, presented in Table 5.

**Figure 10:**
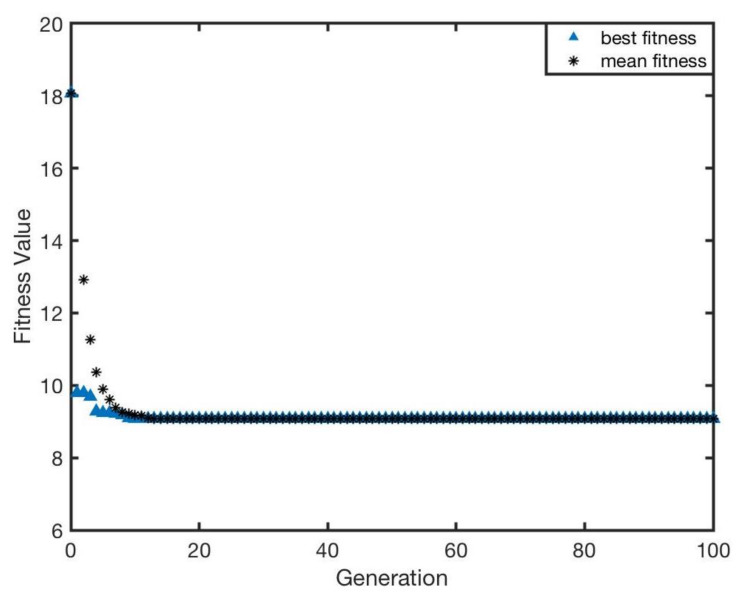
The fitness value of optimisation with respect to generation for IR signal intensities, $\eta_{i}$.

The optimisation performance can be illustrated by the change of fitness function based on Equation (4). The whole optimisation result with respect to generation is presented in Figure 10. It was proved that the genetic algorithm is capable of completely accomplishing the optimisation task. Moreover, as the iteration procedure goes on, the fitness value decreases to approximately $9.05^{\circ }$ that is almost half of the initial value (approximately $18.1^{\circ }$). Furthermore, it can be found that the gradient of descent is extremely steep, which means that optimisation of genetic algorithm can rapidly converge to the optimal solution. It merely experiences roughly 10 iterations to obtain the optimal solution.

**Figure 11:**
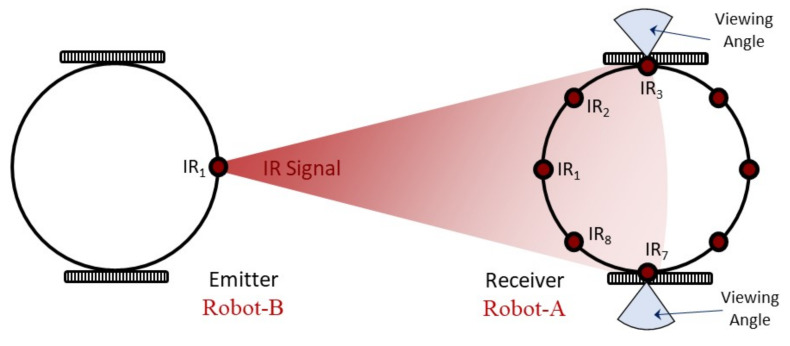
The structure of IR receivers and view angles.

The values of optimised weights for each sensor are presented in Table 5. However, the presumptive range of weights is between 0 and 2. Theoretically, too big or too small weights should not appear in normal condition. Nevertheless, there are two weights $\left(\eta_{3},\eta_{4}\right)$ that are both excessively small. That is because considering the arrangement of sensors on the circuit board of Mona, these two infrared sensors are assigned at the places where receivers’ orientations are not directed to the infrared emitter, with 90${}^{\circ }$ angular distance between the emitter IR 1 and receivers IR 3 and IR 7 separately. Thereby, the infrared signal emitted is out of the detection of infrared receivers due to their viewing angles. The detail of structure is shown in Figure 11. From the diagram, it can be viewed that the IR 3 and IR 7 can hardly receive the infrared signal from the IR 1 emitter of Robot-B.

**Figure 12:**
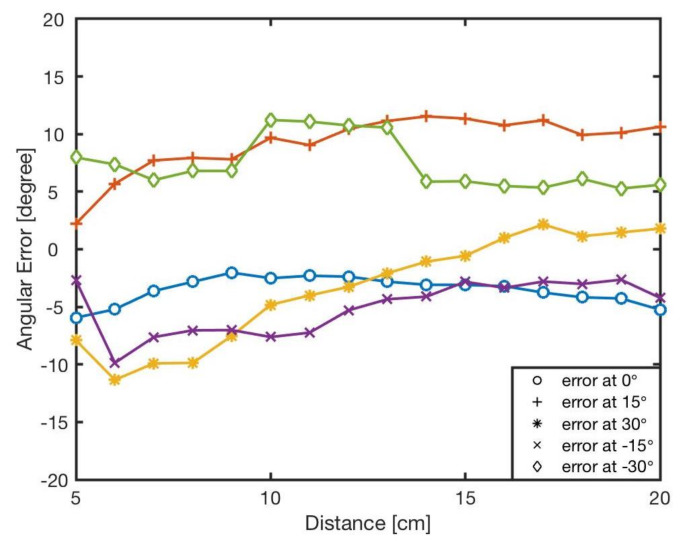
The bearing errors, *e*, at different angles in various distances, $l\in \left[5\text{–}20\right]$ cm after optimising the model parameter, $\eta_{i}$.

**Figure 13:**
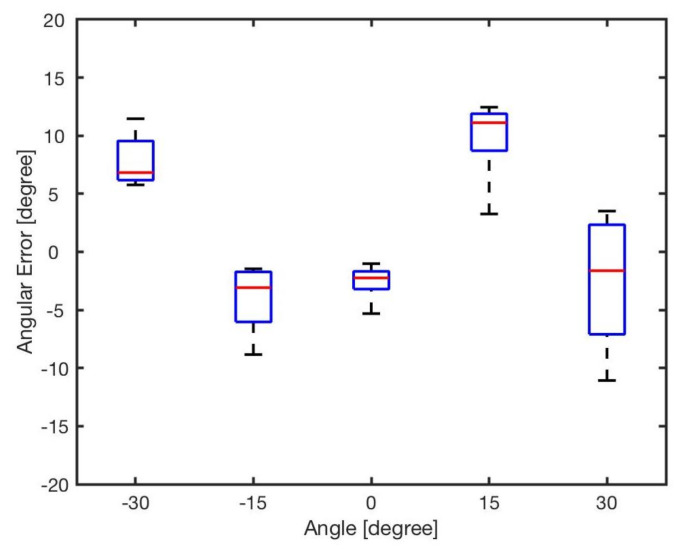
Angular error for different angles after optimising $\eta_{i}$.

Figure 12 illustrates results of the bearing after applying the optimised weights. In general, compared with the original errors without optimisation, the errors at reference angles show a downward trend, with most of errors experiencing about 50% decline and maintaining at approximately $5^{\circ }$. Figure 13 illustrates the angular error for different angles for all the investigated distances. The results show sufficient reduction in total error; however, we can still see the heterogeneity of the IR receivers (sensibility). The average errors with this optimisation for each angle are presented in Table 6. The optimisation results proved that the proposed bearing model can improve the positioning precision for use in swarm robotics applications which mainly do not require a precise positioning.

### 6.3. Redesign of Sensor Configuration

**Figure 14:**
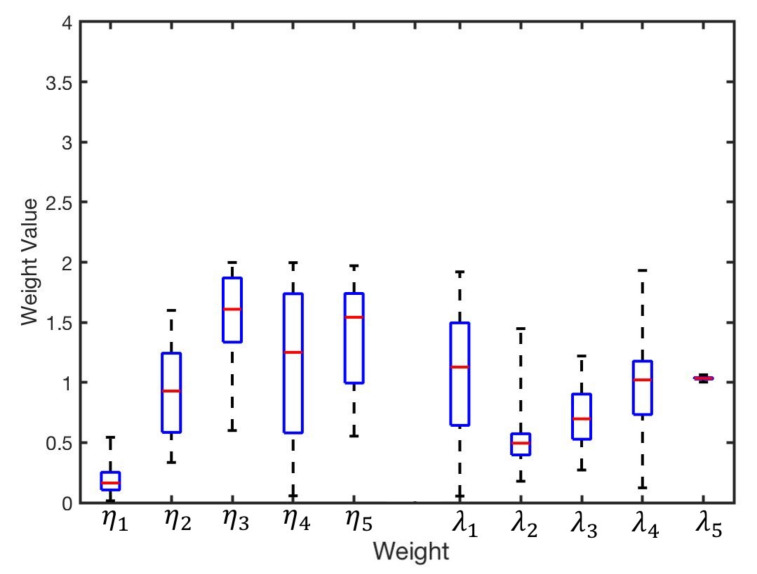
Distribution of the obtained wights of $\eta_{1}\ldots \eta_{5}$ and $\lambda_{1}\ldots \lambda_{5}$ from 100 experiments.

Relocating the sensors’ positions on the circuit board of the robot is another possible method to further eliminate the bearing error and improve the performance. Based on the modified bearing model (3), GA was used to optimise the configuration of infrared sensors. Therefore, the second step of the optimisation focused on 10-dimensional parameters, including $\lambda_{i}$ and $\eta_{i}$. Similar to the first step, the GA optimisation was repeated 100 times to eliminate converging to a local optimum. The distribution of weights is shown in Figure 14.

The “best fitness value” weights for IR intensity and sensor layout are selected, and the optimised result of all the ten-weight values for in the layout is illustrated in Table 7 and Table 8.

**Figure 15:**
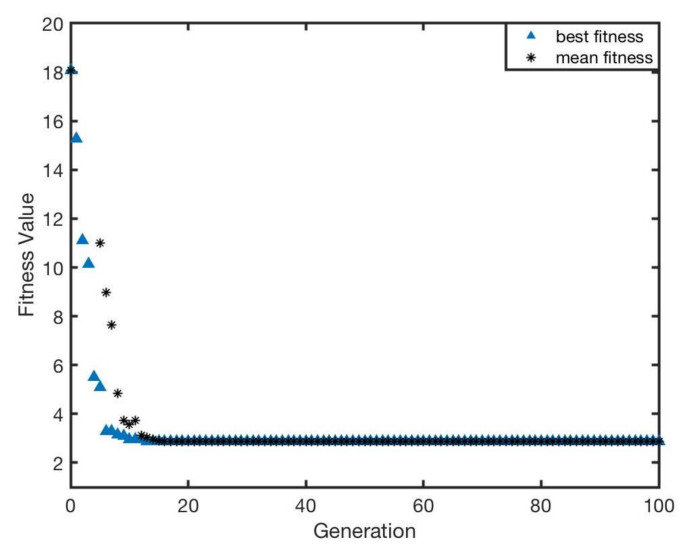
The fitness value of optimisation with respect to generation for IR sensors position and IR intensity, $\lambda_{i}$ and $\eta_{i}$.

The whole optimisation result with respect to generation is presented in Figure 15. GA can achieve the optimisation requirement for sensor layout. After the optimisation, the fitness value declined from 18.1${}^{\circ }$ to approximately 2.8${}^{\circ }$, and the best individual was generated and selected in each generation. The result indicated that the further optimisation to sensor position can acquire better performance (lower fitness value). Therefore, it can be deduced that the adjustment for distribution of sensors (hardware adjustment) would have more significant impact on the improvement of bearing precision.

**Figure 16:**
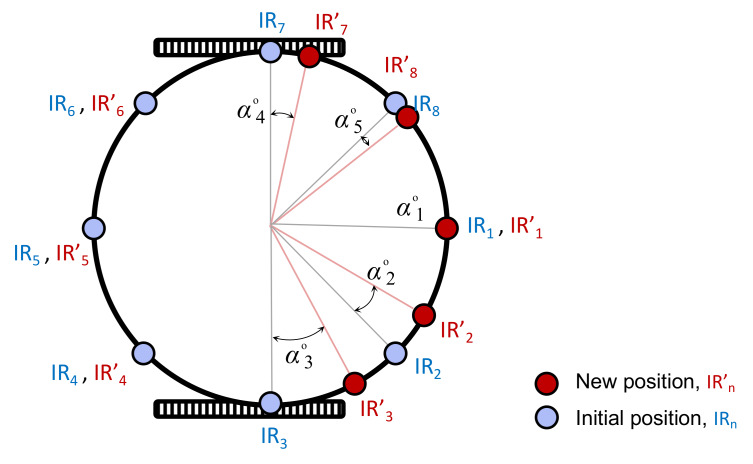
The new sensor layout on the communication board of the robot.

The optimised weights indicate all the sensors’ initial positions need to be readjusted. Based on the proposed mathematical model, new angular distance between each IR sensor and IR${}_{1}$ is calculated by Equation (6). The new sensor layout is presented in Figure 16. Obviously, only the positions of five IR sensors that participated in the bearing experiment will be modified. In addition, the angular distances between the initial positions and the new positions of sensors are calculated by Equation (7). Table 9 reveals the angular displacement, $\alpha_{i}^{\circ }$, in the layout level.

(6) $$\psi_{i_{new}}=\lambda_{i}\psi_{i}$$

(7) $$\alpha_{i}=\left|\psi_{i_{new}}-\psi_{i}\right|\;$$

where $\psi_{i_{new}}$ is the readjusted angular distance between *i*th IR sensor and sensor IR${}_{1}$, and $\alpha_{i}$ indicates the angular displacement between the initial and new sensor position.

**Figure 17:**
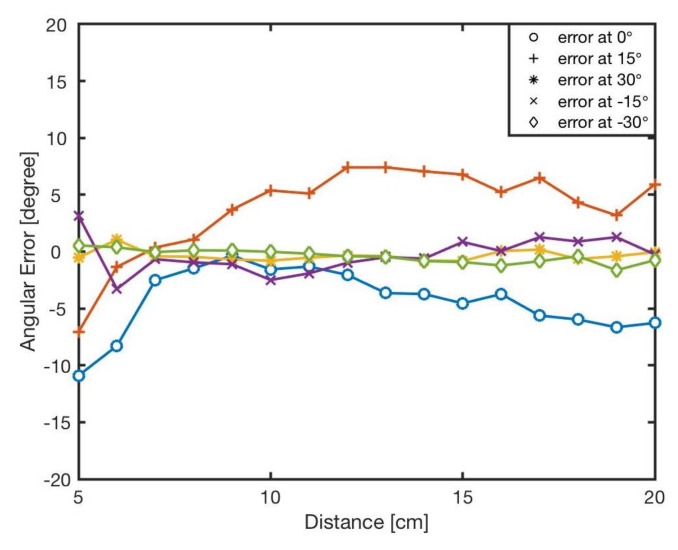
The bearing errors, *e*, at different angles in various distances, $l\in \left[5\text{–}20\right]$ cm after optimising the model parameters, $\lambda_{i}$ and $\eta_{i}$.

Figure 17 shows the results of the bearing after applying optimised weights of $\eta_{i}$ and $\lambda_{i}$. In general, the errors for all of angle cases were significantly reduced.

**Figure 18:**
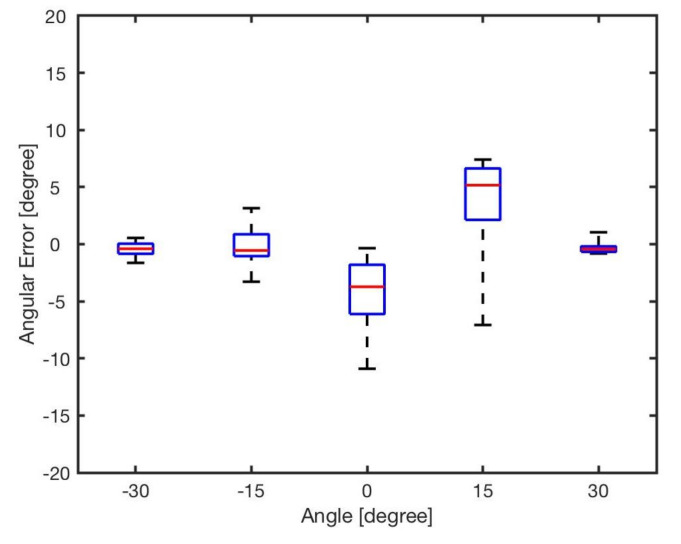
Angular errors for different angles after optimising $\lambda_{i}$ and $\eta_{i}$.

Figure 18 reveals the angular errors after the second phase of the optimisation. Compared to the previous set of optimisation (shown in Figure 13), the results showed a significant reduction of error. Table 10 shows the average error at different angles after optimising both η and λ.

From the results, it can be seen that the further optimisation significantly reduced the bearing errors. The results for experiments with 30${}^{\circ }$, −15${}^{\circ }$ and −30${}^{\circ }$ showed that the bearing errors were almost eliminated. For the experiments with 0${}^{\circ }$ and 15${}^{\circ }$, the average error is lower than 5${}^{\circ }$.

In summary, two phases of optimisation were investigated in this paper. The first optimisation step was applied in the software layer and the second optimisation step investigated the possibility of changes at the hardware level combined with the software layer. Both optimisation steps provided precise bearing estimations. Based on the obtained results, the precision can be improved if we consider displacement of sensors in the hardware layout and at the software level, represented in (3). It must be mentioned that the experiments in this study were implemented using two robots. Hence, it would increase the noise and interference if more neighbouring robots sent IR signals simultaneously. However, it can be solved by (i) reducing the output power of IR emitters to limit them to local, short-range communication [41], (ii) using high-level algorithms for implementing multi-robot serial communication [76] and (iii) utilising sophisticated communication protocols [77]. Although the second set of optimisation further improved the performance, it would be costly to generate the communication boards with the new layout, which is the drawback of this step. Therefore, we will continue employ only optimised η values of the model that was presented in (2) for the future research studies using the real-world Mona robot swarm.

## 7. Conclusions

Aiming to the determine the bearings of a miniature robot with multiple IR sensors in a swarm robotic system, this project proposed a modified mathematical model of bearing estimation based on the existing positioning model, so as to improve the accuracy of positioning when applied into the practical localisation. The developed mathematical model relies on the optimisation to improve accuracy. The specific sequence is to arrange the weights for certain independent variables, and then utilise the GA to acquire the optimal values of these weights that are conducive to eliminating the error of bearing positioning. After optimisation for IR intensity, the fitness value decreased from 18.1${}^{\circ }$ to 9.3${}^{\circ }$, and the average errors at the reference angles were reduced successfully, with the highest extent of decline being 85.7%, and the majority of errors were no more than 5${}^{\circ }$. In addition, after optimisation for sensor layout, the fitness value also experienced a significant reduction to roughly 2.8${}^{\circ }$. For the average errors at different angles, the highest reduction was as high as 99%, and some errors were at a significantly low level, almost close to 0${}^{\circ }$. The results proved that the proposed bearing model is effective and successful for finding the precise bearing of a neighbouring robot in a multi-robot system; e.g., swarm robotics. For future work, we will use the developed module for robotic swarms with large populations. There are several swarm behaviours, such as formation control and foraging, which rely on performance of the bearing estimation.

## Figures and Tables

**Table 1:** List of different miniature robots used in swarm robotics.

Robot	Perception/Communication	Battery	Motion	MCU/MPU	Ref
Colias	IR	LiPo, 3.7 V, 600 mAh	Wheel	ATmega168	[41]
Colias-IV	IR, Bluetooth, RF	LiPo, 3.7 V, 400 mAh	Wheel	ARM Cortex M4	[42]
E-puck	IR, Bluetooth	5 Wh	Wheel	dsPic30	[43]
GRITSBot	IR, RF	LiPo, 3.7 V, 150 mAh	Wheel	ATmega328	[44]
HeRo	IR, WiFi	LiPo 3.7 V, 1 Ah	Wheel	ESP8266	[45]
Jasmine	IR	LiPo, 2 h autonomy	Wheel	ATmega168	[46]
Kilobot	IR	LiPo, 3.4 V, 160 mAh	Vibration	ATmega328	[47]
Kobot	IR, ZigBee	LiPo, 7 Wh	Wheel	PIC16F877A	[48]
MHP	IR	Alkaline, 9 V	Micropump	ARM Cortex M4	[49]
Mona	IR, WiFi	3.7 V, 250 mAh	Wheel	ATmega2560	[8]
r-one	IR, ZigBee	4 h autonomy	Wheel	TI LM3S8962	[50]
S-Bot	IR, WiFi	LiIon 10 Wh	Treels	Intel XScale	[51]
Wanda	IR, ZigBee	LiPo 3.4 V, 250 mAh	Wheel	TI LM3S1960	[52]

**Table 2:** The parameters of genetic algorithm optimisation.

Parameter	Population Size	Chromosome Length	Generation Size	Crossover Rate	Mutation Rate
**Value**	100	100	50	0.6	0.01

**Table 3:** Experimental values or range for variables.

Parameters	Description	Value/Range
φ	Real angular position of neighbouring robot	$\left\{-30^{\circ },-15^{\circ },0^{\circ },15^{\circ },30^{\circ }\right\}$
ϕ	Estimated bearing of neighbouring robot	$\left[-30^{\circ }\text{–}+30^{\circ }\right]\pm 10^{\circ }$
ψ	Angular distance between the IR sensors on PCB	$\left[40^{\circ }\text{–}50^{\circ }\right]$
*l*	Distance between two robots	$\left[5\text{–}20\right]$ cm
λ	Weights for sensors’ angular position	$\left[0\text{–}2\right]$
η	Weights for received IR intensity	$\left[0\text{–}2\right]$

**Table 4:** The average errors for different angles.

Robot Angle, φ	0${}^{\circ }$	15${}^{\circ }$	30${}^{\circ }$	−15${}^{\circ }$	−30${}^{\circ }$
**Average error, $\bar{e}$**	−6.5246${}^{\circ }$	5.7147${}^{\circ }$	−14.2269${}^{\circ }$	−10.4157${}^{\circ }$	5.9967${}^{\circ }$

**Table 5:** The optimal weights for infrared intensity.

Weight Variable	$\eta_{1}$ (IR${}_{1}$)	$\eta_{2}$ (IR${}_{2}$)	$\eta_{3}$ (IR${}_{3}$)	$\eta_{4}$ (IR${}_{7}$)	$\eta_{5}$ (IR${}_{8}$)
**Value**	1.0635	0.9971	0.002	0.0039	1.6833

**Table 6:** The average errors at different angles after optimisation for IR intensity.

Robot Angle	0${}^{\circ }$	15${}^{\circ }$	30${}^{\circ }$	−15${}^{\circ }$	−30${}^{\circ }$
**Average error**	−3.4261${}^{\circ }$	9.2025${}^{\circ }$	−3.4261${}^{\circ }$	−5.1070${}^{\circ }$	7.3875${}^{\circ }$

**Table 7:** The optimal weights for IR intensity.

Weight	$\eta_{1}$ (IR${}_{1}$)	$\eta_{2}$ (IR${}_{2}$)	$\eta_{3}$ (IR${}_{3}$)	$\eta_{4}$ (IR${}_{4}$)	$\eta_{5}$ (IR${}_{5}$)
**Value**	0.0156	1.3529	1.8690	0.1408	1.7361

**Table 8:** The optimal weights for sensors positions.

Weight	$\lambda_{1}$ (IR${}_{1}$)	$\lambda_{2}$ (IR${}_{2}$)	$\lambda_{3}$ (IR${}_{3}$)	$\lambda_{4}$ (IR${}_{7}$)	$\lambda_{5}$ (IR${}_{8}$)
**Value**	0.2717	0.3148	0.9599	1.3060	1.0440

**Table 9:** The displacement between the initial and new sensor layout.

Angular Displacement	$\alpha_{1}$ (IR${}_{1}$)	$\alpha_{2}$ (IR${}_{2}$)	$\alpha_{3}$ (IR${}_{3}$)	$\alpha_{4}$ (IR${}_{7}$)	$\alpha_{5}$ (IR${}_{8}$)
**Value**	0${}^{\circ }$	30.834${}^{\circ }$	3.609${}^{\circ }$	27.54${}^{\circ }$	19.8${}^{\circ }$

**Table 10:** The average errors at different angles after optimisation of η and λ together.

Robot Angle	0${}^{\circ }$	15${}^{\circ }$	30${}^{\circ }$	−15${}^{\circ }$	−30${}^{\circ }$
**Average error**	−4.2877${}^{\circ }$	3.8144${}^{\circ }$	−0.3472${}^{\circ }$	−0.3272${}^{\circ }$	−0.4096${}^{\circ }$

## Abbreviations

The following abbreviations are used in this manuscript:

ADC	Analog–digital converter
GA	Genetic algorithm
PSO	Particle swarm optimisation
ACO	Ant colony optimisation
GPS	Global Positioning System
I^2^C	Inter-integrated circuit
ICP	Iterative closest point
IMU	Inertial measurement unit
IR	Infrared
LiDAR	Light detecting and ranging
PRWS	Proportionate roulette wheel selection
RFID	Radio-frequency identification
SPI	Serial peripheral interface
UAV	Unmanned aerial vehicles
UWB	Ultra-wideband
WLAN	Wireless local area network
